# Tracheobronchomegaly as a Cause of Bronchiectasis in an Adult

**DOI:** 10.1155/2016/5049406

**Published:** 2016-02-28

**Authors:** Vishnukanth Govindaraj, Madhusmita Mohanty Mohapatra, Balla Nagamalli Kumar, Suryakala Narayanasami

**Affiliations:** Department of Pulmonary Medicine, Jawaharlal Institute of Postgraduate Medical Education and Research (JIPMER), Dhanvantri Nagar, Puducherry 605006, India

## Abstract

Mounier-Kuhn syndrome (MKS) is a rare congenital anomaly characterized by dilated trachea and main bronchi due to atrophy of the elastic fibers and smooth muscle cells of the trachea and major airways. Patients of MKS can have varied presentation. The diagnosis is established radiologically and bronchoscopically. There is no specific treatment for MKS. We present an adult man with MKS who presented with recurrent respiratory tract infection. The diagnosis was confirmed by imaging study and fiberoptic bronchoscopy.

## 1. Introduction

Mounier-Kuhn syndrome is a rare congenital anomaly characterized by the presence of dilated trachea and major bronchi. We report a case of this syndrome in an adult male who presented with recurrent respiratory tract infection. Diagnosis was established by high resolution computed tomography (HRCT) of the chest and bronchoscopy.

## 2. Case Scenario

**Figure 1:**
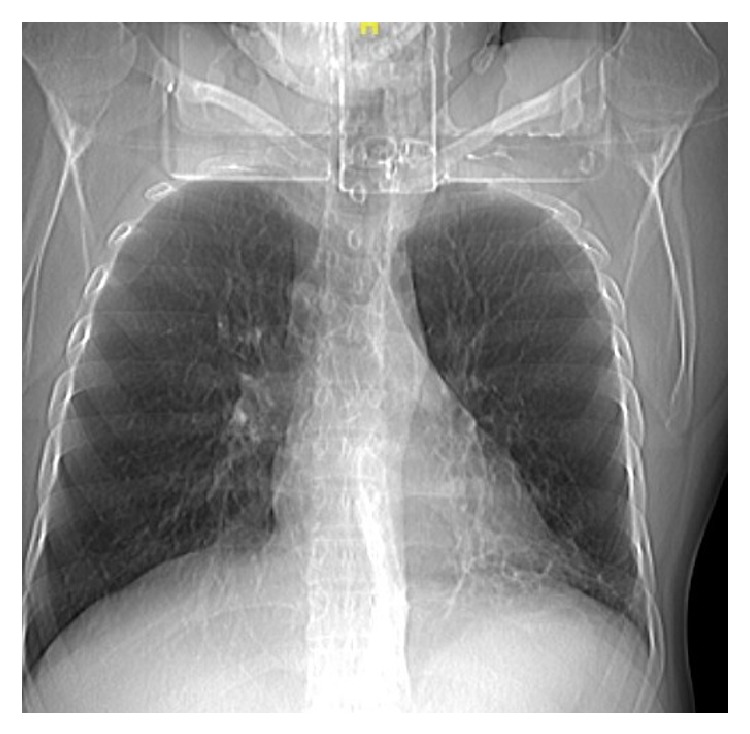
Scanogram showing dilated trachea.

**Figure 2:**
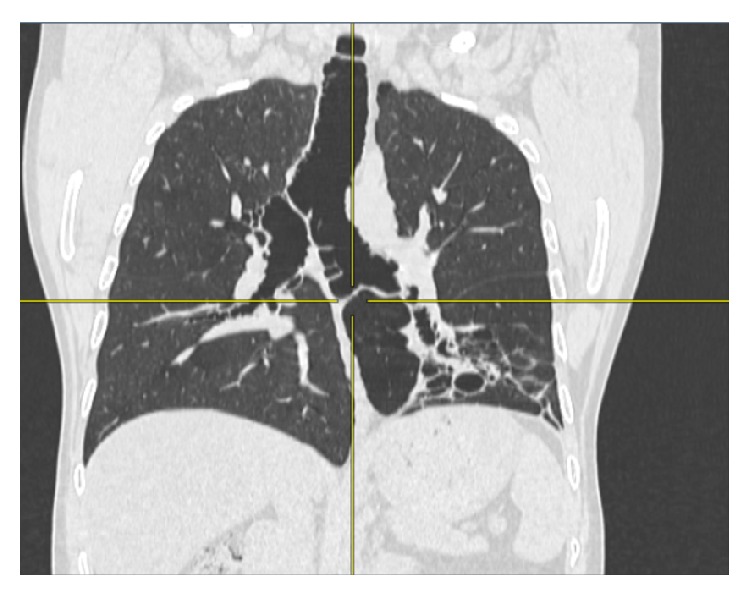
Transverse section of CT showing the grossly dilated trachea and main bronchi.

**Figure 3:**
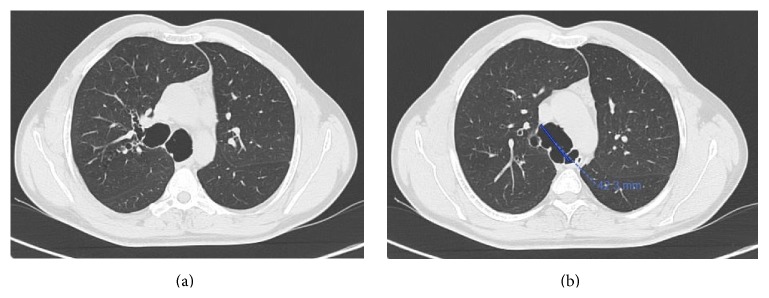
HRCT showing dilated trachea and the main bronchi.

**Figure 4:**
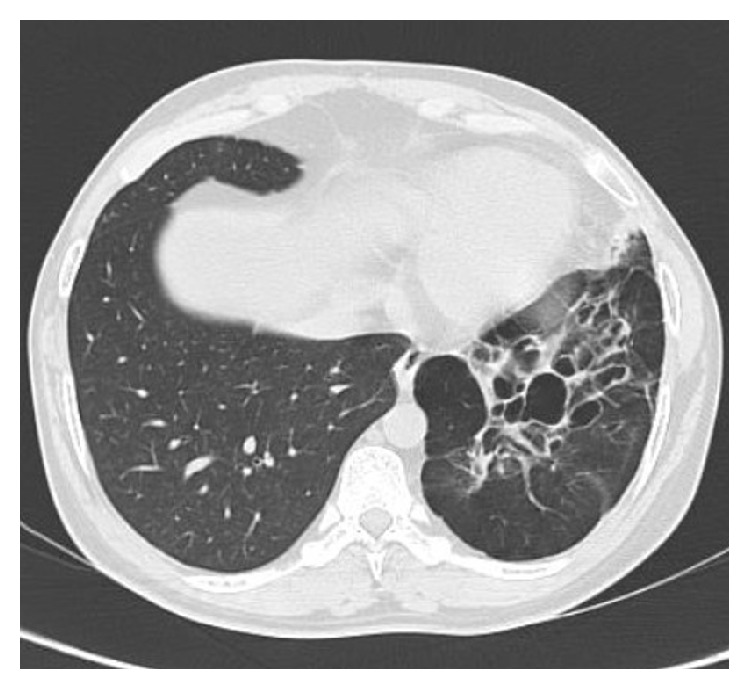
HRCT showing minimal bronchiectatic changes on the left side.

**Figure 5:**
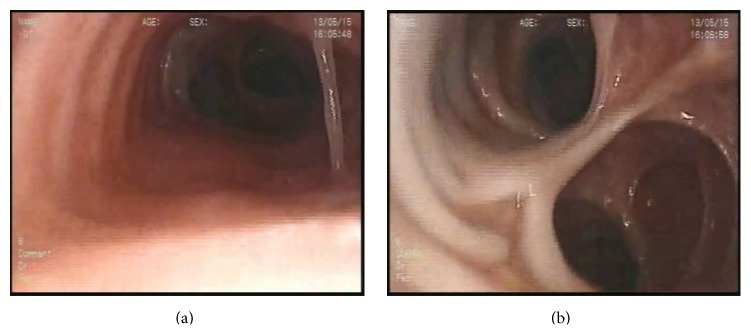
Bronchoscopy images showing dilated trachea and main bronchi.

A thirty-six-year-old agricultural labourer presented with symptoms of cough and expectoration of one-week duration. Respiratory symptoms were present for the last ten years with two to three exacerbations per year. There was no associated breathlessness or wheezing episodes. The exacerbations have been treated symptomatically. He was a never smoker and denied previous tuberculosis history. He is married and has two children. He was born to non-consanguineously married parents. Examination noted normal vital signs, mild ptosis of left eye, and inspiratory crepitations in the left interscapular area. Due to his long standing history, a chest X-ray and serum eosinophil count were performed. Chest X-ray showed presence of retro cardiac cystic shadows with intact and normally positioned left hemidiaphragm. Blood counts were normal. A possibility of left lower lobe bronchiectasis was considered and a high resolution computed tomography (HRCT) scan of the chest was performed. HRCT showed the presence of dilated trachea and both main bronchi, irregularity of the tracheal wall, and few bronchiectatic changes in the left lower lobe. The trachea measured 3.5 cm in diameter and the right and left main bronchi measured 2.5 cm and 2.4 cm, respectively, on cross section (Figures 1, 2, 3(a), 3(b), and 4). A possibility of Mounier-Kuhn syndrome was considered and patient was subjected to bronchoscopy. Flexible bronchoscopy noted dilated trachea and main bronchi with few tracheal protrusions/diverticulae (Figures 5(a) and 5(b)). Cough elicited near total main bronchial collapse. Diagnosis of MKS was established and patient was subjected to further evaluation. Echo cardiography was normal. Pulmonary function test showed a combination of obstruction and restriction pattern. Ophthalmologist opinion was obtained for ptosis and was opined as isolated congenital ptosis. The condition was explained to the patient and his relatives. He is currently on regular visit and undergoes postural drainage and chest physiotherapy at home and his symptoms have not worsened till the last contact.

## 3. Discussion

Mounier-Kuhn syndrome (MKS) also known as tracheobronchomegaly is an uncommon condition characterized by dilatation of the trachea and major bronchi. It is also referred to as trachiectasis, tracheobronchopathia malacia, tracheomegaly, and multiple tracheal diverticula [1]. MKS differs from a closely related condition, William Campbell syndrome. The latter also known as bronchomalacia is characterized by a deficiency of cartilage in the subsegmental bronchi [2].

Mounier-Kuhn syndrome was described by Mounier-Kuhn in 1932 [3]. The syndrome is characterized by dilation of the trachea and bronchi and by recurrent lower respiratory tract infections (LRTIs). The exact aetiology is unknown although the basic pathology is because of atrophy or absence of elastic fibers and smooth muscle cells from trachea down to fourth-order bronchi division [4]. Bronchial and tracheal diverticula can also accompany tracheobronchomegaly. Sarcoidosis, usual interstitial pneumonia, and cystic fibrosis can cause severe fibrosis of the upper lobes, which may result in tracheal enlargement if there is sufficient tracheal traction. Conditions such as Marfan syndrome, Ehler-Danlos syndrome, ataxia telangiectasia, Bruton type agammaglobulinaemia, ankylosing spondylitis, cutis laxa are also associated with secondary tracheobronchial enlargement [5–8]. Mounier-Kuhn syndrome has 3 subtypes. In type 1, there is a minimal symmetric dilation of the trachea and the main bronchi. In type 2 the dilation and diverticula are more distinct. Type 3 is characterized by extension of dilation and diverticula to the distal bronchi [9, 10]. Abdelghani et al. [11] suggested a Clinical Classification Scheme for MKS based on the clinical features. Type 1A consisted of infants who developed MKS after having undergone fetoscopic tracheal occlusion, and Type 1B patients include infants and children who developed MKS after prolonged intubation. Type 2 individuals develop MKS following recurrent pulmonary infections (2A) or pulmonary fibrosis (2B). Type 3 includes patients of MKS with evidence of extrapulmonary elastolysis. Persons with Type 4 MKS have no clear predisposing factors. Our patient had no identifiable predisposing factors.

The condition has a male predominance and usually manifests in middle age. The clinical presentations can vary from asymptomatic patients to respiratory failure. Majority of the patients however have nonspecific symptoms and are usually diagnosed as having chronic bronchitis or bronchiectasis. Symptomatic patients can present with recurrent pneumonia, chronic productive cough, occasional hemoptysis, and progressive dyspnea. Rarely life-threatening hemoptysis, spontaneous pneumothorax may occur. Symptoms occur due to ineffective cough secondary to pathologic dilation in the tracheobronchial tree and the impairment of mucociliary activity leading to difficulty in expectorating secretions and resultant recurrent respiratory infections.

MKS is usually suspected radiologically. Diagnosis is confirmed by computed tomography and bronchoscopy. On CT scan, the diagnosis is made when the transverse diameter of trachea is greater than 3.0 cm and that of right and left main bronchi is more than 2.4 cm and 2.3 cm, respectively. In females, it can be considered to be present when transverse and sagittal diameters of the trachea exceed 21 and 23 mm, respectively, and the diameters of the right and left main bronchi exceed 19.8 and 17.4 mm, respectively [12, 13]. On dynamic radiographic and bronchoscopic imaging the trachea and major bronchi distend with deep inspiration and collapse on expiration. The central airways completely collapse with cough or forced expiration. Tracheal diverticulosis due to protrusion of remnants of the musculomembranous tissue in between the cartilage rings can cause a corrugated or scalloped appearance of trachea as seen in our case. CT chest may also reveal the presence of associated bronchiectasis. Pulmonary function testing may show reduced flow rates, increase in dead space, and increased tidal volume [14]. Bronchoscopy is useful to confirm the diagnosis when CT images are not conclusive.

There is no specific treatment for MKS. Asymptomatic patients usually require no treatment. Smoking cessation is beneficial. Symptomatic patients can be managed by bronchodilators, antibiotics, and chest physiotherapy and postural drainage. The use of night time noninvasive ventilation via continuous positive airway pressure (CPAP) has been tried with success to clear secretions [15]. Tracheal stenting may be beneficial [16]. Few instances of successful laser tracheoplasty to prevent the collapse of posterior membrane of trachea have been reported [17]. Surgery has limited role due to diffuse nature of the condition [18].

## 4. Conclusion

Mounier-Kuhn syndrome (MKS) is probably underdiagnosed as the symptoms are nonspecific. In patients with recurrent respiratory tract infection and bronchiectasis a possible differential diagnosis of MKS should also be considered. A chest X-ray and HRCT of chest can establish or rule out this syndrome.

## References

[1] Rahul S. R., Alok K., Meghwani K. M., Vikas S. (2014). Case report: Mounier-Kuhn syndrome. *Journal of Pulmonary & Respiratory Medicine*.

[2] Konoglou M., Porpodis K., Zarogoulidis P. (2012). Williams-Campbell syndrome: a case report. *International Journal of General Medicine*.

[3] Mounier-Kuhn P. (1932). Expansion of the trachea, radiographic and bronchoscopic findings. *Lyon Medical*.

[4] Schwartz M., Rossoff L. (1994). Tracheobronchomegaly. *Chest*.

[5] Blake M. A., Clarke P. D., Fenlon H. M. (1999). Thoracic case of the day: Mounier-Kuhn syndrome (tracheobronchomegaly). *American Journal of Roentgenology*.

[6] Schoor J. V., Joos G., Pauwels R. (1991). Tracheobronchomegaly: the Mounier-Kuhn syndrome; Report of two cases and review of the literature. *European Respiratory Journal*.

[7] Lazzarini-de-Oliveira L. C., Costa de Barros Franco C. A., Gomes de Salles C. L., de Oliveira A. C. (2001). A 38-year-old man with tracheomegaly, tracheal diverticulosis, and bronchiectasis. *Chest*.

[8] Menon B., Aggarwal B., Iqbal A. (2008). Mounier-Kuhn syndrome: report of 8 cases of tracheobronchomegaly with associated complications. *Southern Medical Journal*.

[9] Himalstein M. R., Gallagher J. C. (1973). Tracheobronchomegaly. *Annals of Otology, Rhinology & Laryngology*.

[10] Payandeh J., McGillivray B., McCauley G., Wilcox P., Swiston J. R., Lehman A. (2015). A clinical classification scheme for tracheobronchomegaly (Mounier-Kuhn syndrome). *Lung*.

[11] Abdelghani A., Bouazra H., Hayouni A. (2009). Mounier-Kuhn syndrome: a rare cause of bronchial dilatation: a case report. *Respiratory Medicine CME*.

[12] Katz I., Levine M., Hermam P. (1962). Tracheobronchomegaly: the Mounier-Kuhn Syndrome. *The American Journal of Roentgenology Radium Therapy and Nuclear Medicine*.

[13] Jain P., Dave M., Singh D. P., Kumawat D. C., Babel C. S. (2002). Mounier-Kuhn syndrome. *Indian Journal of Chest Diseases & Allied Sciences*.

[14] Celik B., Bilgin S., Yuksel C. (2011). Mounier-Kuhn syndrome: a rare cause of bronchial dilation. *Texas Heart Institute Journal*.

[15] Sundaram P., Joshi J. M. (2004). Tracheobronchomegaly associated tracheomalacia: analysis by sleep study. *The Indian Journal of Chest Diseases & Allied Sciences*.

[16] Ernst A., Majid A., Feller-Kopman D. (2007). Airway stabilization with silicone stents for treating adult tracheobronchomalacia: a prospective observational study. *Chest*.

[17] Krustins E., Kravale Z., Buls A. (2013). Mounier-Kuhn syndrome or congenital tracheobronchomegaly: a literature review. *Respiratory Medicine*.

[18] Gupta P., Gorsi U., Bhalla A., Khandelwal N. (2014). Mounier-Kuhn syndrome masquerading pulmonary thromboembolism in an elderly male. *Lung India*.

